# Utilizing Machine Learning and causal graph approaches to Address Confounding Factors in Health Science Research: A Scoping Review

**DOI:** 10.12688/f1000research.159632.2

**Published:** 2025-09-10

**Authors:** Ahmed Hossain

**Affiliations:** 1 Healthcare Management, University of Sharjah, Sharjah, Sharjah, United Arab Emirates; 2 Public Health, North South University, Dhaka, Dhaka Division, 1229, Bangladesh

**Keywords:** public health; bias; confounding; directed acyclic graphs; correlation; causal effects.

## Abstract

Confounding can significantly distort the findings of studies examining cause-and-effect relationships, especially in etiological research. To mitigate this issue, researchers must carefully assess potential confounding variables that may relate to both the exposure and outcome but are not directly influenced by the exposure itself. It is essential that these variables truly impact the outcome rather than simply being correlated with the exposure to avoid false associations. Strengthening confidence in the actual relationship between exposure and outcome requires an understanding of biological mechanisms and the application of various methods to adjust for confounders. The oversight of confounding often arises from inappropriate statistical tests and the aggregation of data across multiple studies. This scoping review article discusses the challenges posed by confounding and presents machine learning approaches for effective control in health science research. Directed acyclic graphs (DAGs) serve as causal graph tools to identify potential confounding variables in health research. By mapping presumed relationships between variables, DAGs enable researchers to estimate causal effects more accurately. While traditional methods such as randomization, matching, and stratification remain effective for controlling confounding, newer techniques like latent variable modeling with negative controls and machine learning methods such as LASSO, Ridge regression, and random forests offer enhanced flexibility and adaptability.

## Introduction

Variables are essential in health research, serving as building blocks for understanding factors influencing health outcomes. They encompass measurable characteristics, attributes, or events that impact health. In health research, distinguishing between dependent and independent variables is fundamental. Often, research involves multiple interacting factors, requiring careful consideration and control to isolate the specific effect of the independent variable on the outcome. For example, in studying the relationship between economic stressors and mental health, variables like economic hardship, financial threat, financial well-being, depression, anxiety, and stress can be considered. ^1^

Identifying and addressing confounding variables is crucial for ensuring the validity and reliability of research findings in health science. Confounding variables can distort the true relationship between the independent and dependent variables, leading to biased results and inaccurate conclusions. ^2^ Failing to account for confounding often occurs due to improper statistical analyses and data aggregation across multiple studies. This emphasizes the pressing need to develop and implement effective control strategies to mitigate the impact of confounding variables in health science research.

Establishing a causal relationship between independent and dependent variables requires careful research design and analysis. Correlation does not imply causation. For example, a study may find a strong association between employment opportunities and mental health symptoms, but this does not necessarily mean that employment causes mental health issues. ^3^ Understanding the distinction between independent and dependent variables is crucial for interpreting research findings in health and other fields. Addressing these challenges ensures the validity and reliability of research findings, which can have significant implications for clinical practice and policy-making.

To minimize the influence of confounding variables, researchers employ various strategies throughout the study design and analysis phases. One key approach is randomization, particularly in clinical trials, where participants are randomly assigned to different intervention groups. ^4^ This method helps distribute confounders evenly among the groups, thereby reducing their potential impact. Another strategy is stratification, which involves dividing the study population into subgroups based on the confounding variable and analyzing these subgroups separately. ^5^ Matching is also a valuable technique where participants with similar confounding characteristics are paired in different study groups to control for those variables. ^6^

Multivariate statistical methods, such as regression analysis, are extensively used to adjust for multiple confounders simultaneously. ^7– 10^ These techniques allow researchers to isolate the effect of the independent variable on the dependent variable while accounting for the influence of confounders. Sensitivity analysis can also be conducted to assess the robustness of study findings to potential confounding. Outcome regression is an innovative way to control for confounding variables by building a statistical model that predicts the outcome variable while accounting for the influence of confounding variables. Standardization involves transforming the data to make the confounding variables comparable across groups.

Implementing control strategies requires careful planning and a thorough understanding of the research context. Researchers must identify potential confounders during the study design phase and select appropriate methods to control them. Transparent reporting of confounder control is crucial for replication and validation. Traditional methods like randomization, matching, and stratification are effective, but newer techniques like latent variable modeling and machine learning offer more nuanced approaches. Directed acyclic graphs can also depict relationships between variables, facilitating unbiased causal effect estimation.

Addressing confounding is vital for the integrity of health science research. By employing robust control strategies, researchers can enhance the accuracy of their findings, contributing to more effective health interventions and policies. As health science evolves, ongoing efforts to refine machine learning methodologies and causal graph approach will be essential in advancing our understanding of complex health phenomena and improving public health outcomes. This scoping review article discussed a few machine learning techniques and causal graph approach to control confounding variables in multivariable data.

## Methods

### Search strategy

We conducted a scoping review of peer-reviewed articles published between January 1, 2010, and December 31, 2023, in PubMed and Google Scholar databases. The search was limited to English-language articles and focused on identifying confounding variables with causal graph and machine learning approaches in health science research. The search combined controlled vocabulary terms (e.g., MeSH) and free-text keywords related to machine learning, causal graphs, confounding, and health sciences. The review adhered to PRISMA guidelines and is given in https://osf.io/krcxt/.

### Selection criteria

This scoping review identified novel methods for addressing confounding variables in health science research: Directed Acyclic Graphs (DAGs) and machine learning techniques (LASSO, Ridge regression, and random forests). Studies were included if they employed these methods for confounder control. Studies were excluded if they did not explicitly address confounding or bias, focused exclusively on simulations without health science applications, or were non-peer-reviewed publications such as editorials, commentaries, or abstracts without full text. Any discrepancies in article selection were resolved by the sole arbiter, AH.

### Study selection process

All identified citations were imported into EndNote for deduplication and management. The study selection process involved a two-phase screening: first of titles and abstracts, followed by a full-text assessment of potentially eligible studies. The entire process, including the reasons for exclusion, was documented using a PRISMA (Preferred Reporting Items for Systematic Reviews and Meta-Analyses) flow diagram.

### Confounding variables

A confounding variable, also known as a confounder, plays a pivotal role in epidemiological studies, influencing the association between the independent variable (the factor under investigation) and the dependent variable (the disease or outcome of interest). ^2, 11^ This additional factor is correlated both with the disease and the independent variable, potentially introducing distortion or masking the true effects of the primary variable on the disease being studied. ^12^ Confounding factors in a study are not restricted to variables directly impacting both the exposure and outcome; they can exhibit diverse relationships with the exposure and outcome. Such as,

1. A confounder can influence the exposure and the outcome directly.
2. It can affect the exposure and be influenced by another factor that affects the outcome.
3. Alternatively, it can affect the outcome and be influenced by another factor that affects the exposure.

The article “On the definition of a confounder” by VanderWeele and Shpitser (2014) clarifies the concept of confounding and introduces the term “surrogate confounder”. ^13^ For example, one studying caffeine intake and heart disease risk failed to consider physical activity, a known confounder. Since individuals who exercise regularly tend to consume more caffeine and have a lower heart disease risk, physical activity serves as a surrogate confounder in the study. However, using physical activity level as a surrogate for the unmeasured confounder may not fully address the confounding effect, leading to potential bias in the study results.

**Figure 1.**
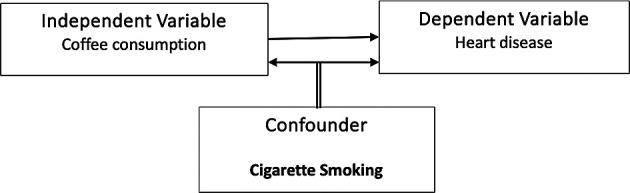
Example of a confounding variable.

To illustrate a confounding variable, as shown in Figure 1, consider a hypothetical scenario where a researcher investigates the association between **coffee consumption** and **heart disease**. The initial hypothesis suggests that coffee drinkers might have a higher prevalence of heart disease compared to coffee non-drinkers. However, a confounding variable, such as **smoking**, could distort the relationship. It is observed that coffee drinkers in the study also tend to smoke more cigarettes than coffee non-drinkers. Consequently, smoking becomes a confounding variable in this analysis, creating ambiguity about whether the increased heart disease risk is genuinely associated with coffee consumption or if it is attributable to the confounding variable of smoking.

This situation underscores the complexity of untangling causation in epidemiological studies, especially in the absence of experimental designs. Due to various constraints like technical, ethical, or financial considerations, researchers often rely on observational studies in public health. Understanding and accounting for confounding variables are critical in these studies to draw accurate and meaningful conclusions about causal relationships. It is essential to conduct meticulous epidemiological studies, carefully considering potential confounders, to inform the development of effective preventive measures in public health.

Here are some more examples of confounding variables:

1. While smoking is strongly associated with ischemic heart disease, income level can be a confounder. High-income individuals might have better access to healthcare and healthier lifestyles, leading to lower heart disease rates, even if there is a positive association between income level and smoke.
2. Research exploring the impact of exercise on depression could be confounded by social support, as individuals with stronger social support are more likely to exercise and have lower depression rates. Social support provides encouragement, motivation, and positive reinforcement, which can help individuals initiate and maintain regular exercise routines.
3. Studies investigating the link between diet and lung cancer risk may face confounding from smoking, as smokers are more likely to have an unhealthy diet and a higher risk of lung cancer. Smoking is associated with other unhealthy lifestyle behaviors, such as poor diet, alcohol consumption, and sedentary lifestyle, these factors may confound the association between diet and lung cancer risk.

## Effect of confounding variables in health research

Confounding variables pose a significant challenge in health research by influencing both the independent and dependent variables, potentially distorting the observed relationships, and leading to misleading results, either falsely positive or falsely negative. ^14^ Upward and downward confounding refer to the direction of bias that occurs when an extraneous variable is not adequately controlled for in a study. ^8^

Upward confounding: Upward confounding occurs when a positively associated confounding variable overestimates the effect of the exposure on the outcome, leading to an inflated observed association. ^15^ In other words, the observed association between the exposure and the outcome appears stronger than it actually is because the confounding variable artificially inflates the effect size. Failure to account for the confounder leads to an overestimation of the true association between the exposure and the outcome.

Downward confounding: Downward confounding arises when a negatively associated confounding variable underestimates the impact of the exposure on the outcome, resulting in a weakened observed association. ^16^ Failure to adequately address this confounder can obscure or underestimate the true relationship between the exposure and the outcome.

In epidemiology, when discussing the relationship between exposure and outcome, some factors are considered “on the causal pathway.” This means that these factors are intermediate steps through which the exposure leads to the outcome. Adjusting for these factors in statistical analyses could essentially remove the effect of interest because they are part of the sequence of events linking the exposure and outcome.

To illustrate with an example, let’s consider smoking as an exposure and lung cancer as the outcome variable. If we view chronic cough as an intermediate step along the causal pathway between smoking and lung cancer, adjusting for chronic cough in the analysis could potentially obscure the genuine association between smoking and lung cancer. This is because chronic cough is influenced by smoking and, consequently, plays a role in the development of lung cancer.

In summary, when some factors are part of the causal pathway, adjusting for them in statistical analyses may not be appropriate as it can alter the interpretation of the relationship between exposure and outcome.

## Machine learning approaches for confounding control

New techniques like latent variable modeling with negative controls, inverse probability of treatment weighting (IPTW) and g-estimation offer more flexibility in applying standardization for confounding control. ^17^ The method introduces a latent variable to represent unobserved confounding factors and assumes that these factors affect both the exposure and the negative control (a variable unrelated to the outcome) to the same degree.

Machine learning techniques like Least Absolute Shrinkage and Selection Operator (LASSO), Ridge regression, and random forests play a pivotal role in identifying confounding variables and mitigating bias, especially in large healthcare datasets where unmeasured confounding may exist. ^18^ The article also noted that hybrid methods, combining traditional techniques like stepwise regression, directed acyclic graphs, and knowledge-based approaches with machine learning, showed promising results.

LASSO applies a penalty to the model’s coefficients, resulting in shrinkage and potentially reducing some coefficients to zero. ^19^ This sparsity characteristic simplifies the model and enhances interpretability by retaining only the most relevant features. Ridge regression also employs regularization to prevent overfitting but differs from LASSO in that it does not reduce coefficients to exactly zero, making it a more stable option when managing highly correlated features. ^20^

Random Forests, on the other hand, evaluate the significance of each feature (or confounder) in predicting the outcome. ^21^ They excel at capturing complex, non-linear relationships between variables and are robust against outliers and noise in the data. By employing these diverse strategies, researchers can enhance the reliability and robustness of their findings in health science research.

Machine learning should be viewed not as a replacement for causal theory but as a powerful complement to it. While traditional methods remain sufficient and more interpretable for simpler problems, the growing complexity and high dimensionality of modern data make ML-based approaches, when embedded within a causal framework, more effective for addressing confounding and producing robust causal estimates. The focus is therefore shifting from a dichotomy of ‘ML versus traditional methods’ to the challenge of optimally integrating ML into the causal inference pipeline.

## Identifying confounders by causal graphs

Causal graphs, also known as directed acyclic graphs (DAGs), provide a visual representation of the causal relationships between variables in a given system or phenomenon. ^22^ In the context of evaluating potential confounding bias and other biases in epidemiological studies, causal graphs serve as a gold standard tool. ^23^ By visually mapping out the relationships between variables, including exposure, outcome, and potential confounders, causal graphs allow researchers to assess the likelihood of confounding and other biases affecting their study results.

Researchers can use causal graphs to identify variables that may act as confounders, mediators, or moderators in the relationship between the exposure and outcome of interest. By including these variables in their analyses or adjusting for them appropriately, researchers can control for potential sources of bias and obtain more accurate estimates of the true causal effect. However, DAGs don’t usually show how variables might influence each other indirectly (interactions). This is because they focus on the overall structure of relationships, not the specific details of how strong or curved those relationships might be.

**Figure 2.**
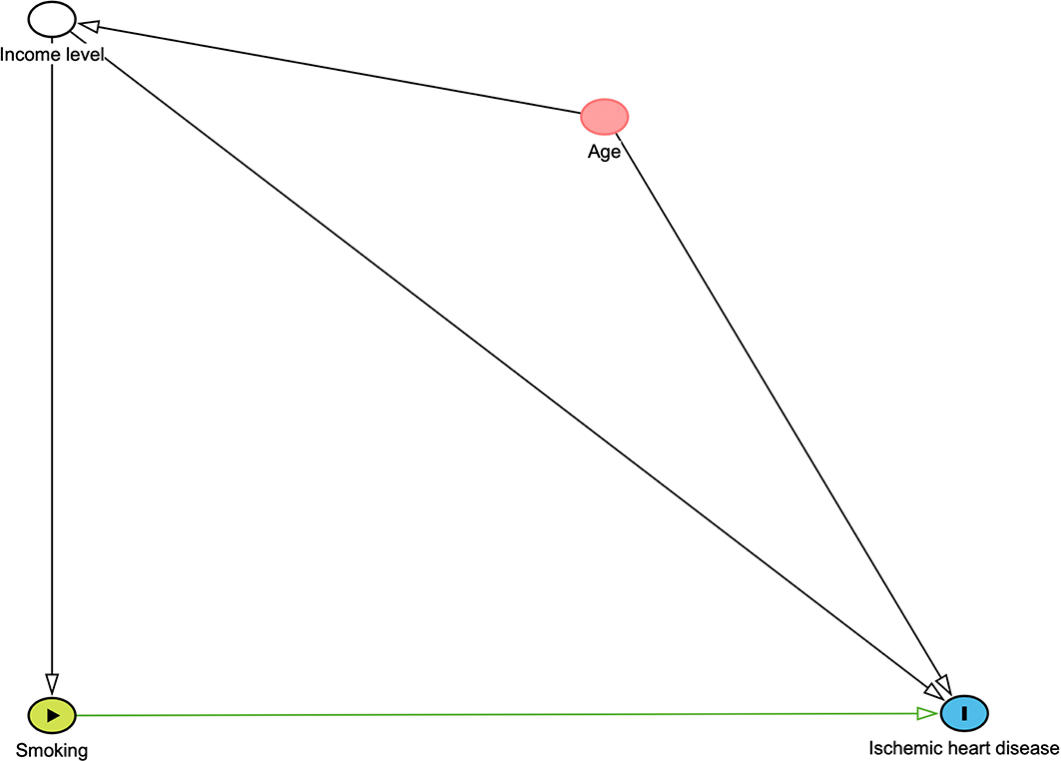
DAG demonstrating causal relationships and potential biasing pathways affecting the association between smoking and ischemic heart.

We investigated a hypothetical DAG to adjust potential confounders in investigating the relationship between smoking and ischemic heart disease. This figure was constructed through DAGITTY ( http://www.dagitty.net/dags.html#) and is given in Figure 2. This graphical method depicts hypothesized causal relationships and deduces the statistical associations implied by these causal relationships. We consider two potential confounders income level and age. The minimally sufficient adjustment set is the combination of the fewest nodes that, being ancestors of both the exposure and outcome. In this conceptual diagram, each circle represents an individual exposure (‘node’) of theoretical relevance to this hypothesis; each node is interconnected by directional arrows (‘edges’) that represent theoretical associations based on the researchers’ assessment of a priori literature and determination of biological plausibility. Smoking was the main exposure of interest (green node with black border), with ischemic heart disease (blue node with black border) as the outcome of interest. In this instance, all the other exposures (‘nodes’) are theoretically causally associated with (i.e., ancestors of ) both the exposure and the outcome. These ‘adjusted variables’ can then be introduced into the multivariate modelling as potential confounders. In this example, minimal sufficient adjustment is containing Income level in the model for estimating the total effect of Smoking on Ischemic heart disease.

## Identifying confounders using change of an effect size

Historically, researchers used the change in estimate method to identify confounders by observing how the effect size of an exposure changes when potential confounders are adjusted for in the analysis. If the effect size changed substantially, it was considered evidence of confounding. ^24– 26^ Calculating odds ratios in the context of confounding variables involves examining how the association between an exposure and an outcome change when considering the influence of a third variable. The odds ratio (OR) is a statistical measure used in epidemiology and other research fields to assess the strength and direction of association between two categorical variables. It is commonly employed in case-control studies and logistic regression analyses. The odds ratio is calculated as the ratio of the odds of an event occurring in one group to the odds of the same event occurring in another group. Here are a few examples illustrating the impact of confounding variables on odds ratios:

Example 1: Consider a scenario where a research study explores the link between smoking (exposure) and ischemic heart disease (outcome). However, age emerges as a potential confounding variable since older individuals are more likely to both smoke and develop ischemic heart disease. To ascertain whether age indeed acts as a confounding variable, odds ratios are calculated both with and without considering age.

• **Without considering age:** The odds of developing lung cancer among smokers are compared to the odds among non-smokers. Let’s assume the calculated value is 5.0, signifying that smoking is associated with a fivefold increase in the risk of lung cancer.
• **After considering age:** A logistic regression model is applied, incorporating age as a variable. The resulting adjusted odds ratio is determined to be 3.0.

If the odds ratio decreases after adjusting for age, this signals that age plays a confounding role, influencing the association between smoking and lung cancer. In this context, the reduction from 5.0 to 3.0 indicates that age was indeed a confounding variable impacting the observed relationship between smoking and lung cancer.

Example 2: Suppose research explores the association between regular exercise (exposure) and obesity (outcome). Socioeconomic status (SES) is a confounding variable, as it is linked to both exercise habits and obesity. Now to investigate whether age is a confounding variable we will have to calculated Odds Ratio with and without considering SES variable.

• **Without considering SES:** The odds of experiencing obesity among those who engage in regular exercise are compared to the odds among those who don’t exercise regularly. Let’s assume the calculated value is 0.7, indicating that regular exercise is associated with a 30% reduction in the risk of weight gain.
• **After considering SES:** After applying a logistic regression model with SES adjustment we found the odds ratio is 1.2.

If the odds ratio undergoes significant change after adjusting for SES, it implies that SES functions as a confounding variable affecting the association between exercise and obesity. In this context, a substantial change in the odds ratio from 0.7 to 1.2 suggests that SES indeed played a confounding role, influencing the observed relationship between exercise and obesity.

However, researchers are advised against relying on the change in estimate method to identify confounders, especially when dealing with non-collapsible measures like the odds ratio. Non-collapsible measures can introduce bias in estimating the association due to inconsistent effects of confounder adjustment across different strata. Hence, alternative methods or approaches are recommended for identifying and adjusting for confounding in statistical analyses.

## Confounder control: Elimination vs. Inclusion

Controlling for confounders in research is crucial to ensure meaningful conclusions from the data. ^26^ Two main approaches exist: elimination and inclusion. Both have their strengths and weaknesses, so choosing the right one depends on specific study and data.

### Confounder elimination

The method involves excluding or restricting individuals with confounding characteristics from the analysis. ^27^ For Example, in studying the relationship between smoking and developing lung cancer, it is possible to eliminate individuals with older adults that could affect developing lung cancer. It is simple to implement, reduces potential confounding bias. In another hypothetical study, examining the association between smoking and ischemic heart disease in community adults, age could act as a confounder. To control for age-related confounding during study design, a straightforward approach would be to implement restriction. This might involve limiting the study to adults aged 60 years and older. While restriction can partially address confounding by age, it may limit the generalizability of study findings to other groups.

However, it can lead to smaller sample sizes, reducing generalizability and power. Moreover, it may not eliminate all relevant confounders, potentially missing important effects.

### Confounder inclusion

The method includes adding confounding variables as additional predictors in your statistical model. ^28^ For example, in the exercise and weight loss study, we may include variables like income level and access to healthy food alongside exercise in the regression analysis.

The multivariable regression analysis can control for multiple confounders simultaneously, potentially revealing more nuanced relationships. ^29, 30^ Moreover, a latent variable strategy with negative controls helps account for hidden factors affecting both exposure and outcome, leading to more accurate estimates of how prenatal factors influence outcomes. ^17^

Another example, in a clinical trial testing the effectiveness of a new drug, if researchers suspect that age may confound the results, they could control by inclusion by considering age as an independent variable. Participants might be divided into different age groups, and the impact of the drug could be assessed within each age group separately. This way, the potential influence of age on the results is explicitly considered.

One strength of this method lies in its ability to maintain larger sample sizes, enhancing generalizability and statistical power. However, its successful application necessitates the careful selection of relevant confounders. Additionally, this method demands more intricate analysis, potentially requiring advanced statistical techniques.

### Limitations of the study

This study includes inclusion and exclusion criteria determined by a single author, introducing the potential for subjectivity and variability in study selection, as well as researcher bias. This review discusses an overview of the literature rather than an in-depth, critical analysis of individual studies, which limits the depth of evaluation. Additionally, this scoping review does not involve any detailed analysis, restricting its ability to draw definitive conclusions about specific models. The study also did not explore alternative approaches, such as elastic net and boosting, for managing confounding variables, which could have enriched its scope. Despite these limitations, this scoping review provides a valuable foundation for further research, including systematic reviews focused on machine learning techniques for addressing confounding variables. By acknowledging these limitations and adopting rigorous methodological strategies in future studies, researchers can enhance the impact and utility of scoping reviews in this field.

## Conclusion

Understanding confounding is crucial for accurate causal inference. By grasping its definitions, control mechanisms, and connections to exchangeability and collapsibility, researchers can design and analyze studies rigorously. To address confounding, meticulous evaluation of potential confounding variables is essential, ensuring they meet all criteria for adjustment without introducing bias. While correlation with the exposure is important, establishing their true influence on the outcome is equally vital to avoid spurious associations. Biological mechanisms and sensitivity analyses can provide valuable insights into the stability of results under different adjustment strategies. Careful assessment and control of confounding in epidemiological studies are crucial for ensuring the accuracy of estimated exposure-outcome associations. Directed acyclic graphs (DAGs) are powerful visual tools in health research. By mapping relationships between variables, DAGs help identify potential confounding factors, leading to more accurate estimates of causal effects. While traditional methods like randomization, matching, and stratification remain valuable, newer techniques like latent variable modeling with negative controls offer even greater flexibility in controlling for confounding. Future research should focus on developing frameworks that seamlessly integrate machine learning with causal inference, ensuring both methodological rigor and interpretability. Emphasis is needed on creating guidelines for selecting when ML offers advantages over traditional approaches, designing hybrid methods that leverage the strengths of both, and validating these approaches across diverse, high-dimensional health datasets. Building user-friendly tools and fostering interdisciplinary collaboration will be essential to translate these advances into practical applications for health science research.

## Data Availability

No data associated with this article. OSF: PRISMA-P and PRISMA-ScR checklist for “Utilizing Machine Learning and causal graph approaches to Address Confounding Factors in Health Science Research: A Scoping Review.” https://doi.org/10.17605/OSF.IO/KRCXT. ^31^ The project contains the following Reporting guidelines data:
• PRISMA-ScR-Fillable-Checklist_AH.docx Data are available under the terms of the Creative Commons Attribution 4.0 International license (CC-BY 4.0).
