# Supplementary material for: Utilizing Machine Learning and causal graph approaches to Address Confounding Factors in Health Science Research: A Scoping Review
Source: F1000Res. 2025 Sep 10;14:129. Originally published 2025 Jan 27. [Version 2] doi: 10.12688/f1000research.159632.2 (PMC12457880; doi:10.12688/f1000research.159632.2)
Supplement: Peer review history

# Reviewer report: Reviewer response for version 2

**DOI:** 10.5256/f1000research.187911.r411686

**Published:** 2025-09-23

**Authors:** Franklin Akwasi Adjei

**Affiliations:** 1 University of Wyoming, Wyoming, USA

The manuscript presents a comprehensive scoping review of methods that are used to identify and control confounding variables in health-related research. The rationale for this review is clearly articulated throughout the manuscript. A confounding variable, which is the distortion of the observed relationship between an exposure and an outcome by an external variable, is a significant challenge in observational and experimental studies. The authors stated the need for addressing confounding to avoid biased conclusions, emphasizing that failure to control confounders can lead to both falsely positive and falsely negative results. The review's objectives are clearly stated, supported by practical examples and hypothetical scenarios, such as the relationship between smoking, coffee consumption, and heart disease. These examples are both practical and common, justifying the study's objectives.

 The manuscript has given sufficient methodological detail to allow replication of the review. The author describes the search strategy comprehensively, including the databases they used and the publication times. The criteria for including and excluding articles were also listed, and PRISMA guidelines were followed. Additionally, the manuscript details the statistical approaches used in the included studies, providing explanations that enable readers to understand, reproduce, and critically evaluate the analytical methods.

 The statistical analysis and interpretation presented in the manuscript are appropriate and aligned with the objectives of the review. The authors correctly explain the concepts of confounding variables and the implications of failing to adjust for these variables in health-related studies, such as epidemiological ones.

 The review's conclusions are well supported by the evidence and discussion. The manuscript highlights the importance of accurately identifying confounders, assessing their effects on both exposure and outcome variables, and using suitable control strategies. The authors also recognize limitations, such as potential subjectivity in study selection, the focus on a scoping rather than an in-depth review, and the omission of some machine learning methods that could offer further insights.

 As a remark, this scoping review has certain limitations that could have been addressed, notably the variability among the included studies. Differences in population characteristics, health outcomes, study designs, and statistical methods make direct comparisons difficult and prevent drawing broad conclusions. Additionally, machine learning and causal inference techniques, including deep learning, causal forests, and boosting, are evolving rapidly, and this may have led to some recent advances being overlooked, thereby creating gaps in the review’s scope. A single author carried out the selection process, which can lead to potential bias or subjectivity in choosing studies. Lastly, since the review broadly covers health science research, the relevance of specific methods can vary across fields like epidemiology, clinical trials, or health services research. Thus, some recommendations may not be fully applicable across all health research domains, limiting their overall generalizability and usefulness.

 Despite these constraints, the conclusions are grounded in the reviewed evidence, and this makes sure that there is scientific credibility.

Are the rationale for, and objectives of, the Systematic Review clearly stated?

Yes

Is the statistical analysis and its interpretation appropriate?

Yes

If this is a Living Systematic Review, is the ‘living’ method appropriate and is the search schedule clearly defined and justified? (‘Living Systematic Review’ or a variation of this term should be included in the title.)

Not applicable

Are sufficient details of the methods and analysis provided to allow replication by others?

Yes

Are the conclusions drawn adequately supported by the results presented in the review?

Yes

Reviewer Expertise:

Public health, environmental health

I confirm that I have read this submission and believe that I have an appropriate level of expertise to confirm that it is of an acceptable scientific standard.

# Reviewer report: Reviewer response for version 1

**DOI:** 10.5256/f1000research.175391.r411684

**Published:** 2025-09-08

**Authors:** Vipin Vageriya

**Affiliations:** 1 Charotar University of Science and Technology, Changa, Gujarat, India

Casual graph approach is important to establish/check the relationship between two variables. DAGs are generally used to model dependencies on variables and workflows, such as task scheduling and data processing. The objective of study is clear. The study conclusion is also significant. I personally believe, it is good paper. The example cited by author are also self-explanatory and easy to understand.

Are the rationale for, and objectives of, the Systematic Review clearly stated?

Yes

Is the statistical analysis and its interpretation appropriate?

I cannot comment. A qualified statistician is required.

If this is a Living Systematic Review, is the ‘living’ method appropriate and is the search schedule clearly defined and justified? (‘Living Systematic Review’ or a variation of this term should be included in the title.)

Yes

Are sufficient details of the methods and analysis provided to allow replication by others?

Yes

Are the conclusions drawn adequately supported by the results presented in the review?

Yes

Reviewer Expertise:

Child Epilepsy, Growth and Development, Parenting, Child Psychology

I confirm that I have read this submission and believe that I have an appropriate level of expertise to confirm that it is of an acceptable scientific standard.

# Response

**Published:** 2025-09-08

**Authors:** Ahmed Hossain

Thanks

# Reviewer report: Reviewer response for version 1

**DOI:** 10.5256/f1000research.175391.r368463

**Published:** 2025-03-11

**Authors:** Ali Husnain

**Affiliations:** 1 Chicago State University, Chicago, Illinois, USA

This scoping review examines the challenges of confounding in health science research and discusses how machine learning and causal graph approaches can mitigate confounding bias. It highlights traditional confounder control methods such as randomization, matching, and stratification and introduces modern techniques, including directed acyclic graphs (DAGs), LASSO regression, Ridge regression, and Random Forests. The paper emphasizes that effective control of confounding is crucial for ensuring the validity of causal inferences in epidemiological studies.

 The author conducted a scoping review of peer-reviewed articles published between 2010 and 2023, using PubMed and Google Scholar. The study follows PRISMA guidelines, with an explicit focus on identifying confounding variables through machine learning models and causal graphs. However, the selection process was conducted by a single reviewer, which introduces potential bias. The paper concludes that emerging computational approaches offer flexibility and adaptability in confounder control but acknowledges that further validation and comparative research are necessary.  This article presents a valuable discussion on confounder control using machine learning and causal graphs in health science research. However, to improve scientific rigor and reproducibility, the following revisions are necessary:

1. **Enhance methodology transparency** by detailing the search strategy, inclusion/exclusion criteria, and study selection process.
2. **Improve statistical rigor** by adding comparative analyses and empirical evidence on machine learning methods versus traditional confounder control approaches.
3. **Strengthen discussion and conclusions** by providing clear recommendations for researchers and identifying future research directions.

Addressing these concerns will significantly improve the paper’s credibility and contribution to the field.

**Overall Rating: Revise and Resubmit**

Are the rationale for, and objectives of, the Systematic Review clearly stated?

Yes

Is the statistical analysis and its interpretation appropriate?

Partly

If this is a Living Systematic Review, is the ‘living’ method appropriate and is the search schedule clearly defined and justified? (‘Living Systematic Review’ or a variation of this term should be included in the title.)

Not applicable

Are sufficient details of the methods and analysis provided to allow replication by others?

Partly

Are the conclusions drawn adequately supported by the results presented in the review?

Partly

Reviewer Expertise:

Epidemiology & Public HealthMachine Learning in HealthcareBiostatistics & Causal InferenceSystematic Review Methodologies

I confirm that I have read this submission and believe that I have an appropriate level of expertise to confirm that it is of an acceptable scientific standard, however I have significant reservations, as outlined above.
